# Pharmacogenetic markers to predict safety of antipsychotics in adolescents experiencing acute psychotic episodes

**DOI:** 10.1192/j.eurpsy.2022.219

**Published:** 2022-09-01

**Authors:** D. Ivashchenko, K. Akmalova, E. Grishina, Y. Shevchenko, D. Sychev

**Affiliations:** 1 Russian Medical Academy of Continuous Professional Education, Child Psychiatry And Psychotherapy, Moscow, Russian Federation; 2 Russian Medical Academy of Continuous Professional Education, Molecular Medicine, Moscow, Russian Federation; 3 Russian Medical Academy of Continuous Professional Education, Clinical Pharmacology And Therapeutics, Moscow, Russian Federation

**Keywords:** Adolescents, Safety, Antipsychotics, pharmacogenetics

## Abstract

**Introduction:**

There are relatively fewer pharmacogenetic studies of antipsychotics in adolescents than in adult patients. The development of personalized pharmacotherapy is promising.

**Objectives:**

Identify the most significant pharmacogenetic predictors of antipsychotic safety in adolescents experiencing acute psychotic episodes

**Methods:**

The study included 101 adolescents diagnosed with acute polymorphic psychotic disorder at the time of admission (F23.0-9 according to ICD-10). All patients were taking an antipsychotic as their main treatment for 14 days. Children’s Global Assessment Scale (CGAS), Positive and Negative Symptoms Scale (PANSS), Clinical Global Impression Severity (CGI-S) and Improvement (CGI-I), UKU Side Effects Rating Scale (UKU SERS), Sympson-Angus Scale (SAS), Barnes Akathisia rating scale (BARS) were used. All study participants underwent pharmacogenetic testing of pharmacokinetic and pharmacodynamic factors.

**Results:**

CYP2D6 “intermediate” metabolism increased the risk of developing an adverse reaction by a trend of significance (OR=2.616 (95% CI 0.950-7.203); p=0.063). Carriage of HTR2A rs6313 was associated with a lower score on the UKU SERS “Other Symptoms” subscale (Beta=(-0.289); p=0.003) and an objective score on the BARS akathisia severity scale (Beta=(-0.217); p=0.029). DRD3 rs324026 carriers had a lower BARS akathisia scale score (Beta=(-0.349); p=0.004); DRD3 rs6280 carriers had a lower SAS extrapyramidal symptom severity scale score (Beta=(-0.351); p=0.003). Carriers of ANKS1B rs7968606 were associated with a higher SAS scale score (Beta=0.237; p=0.017).

**Conclusions:**

We proposed that genotyping of CYP2D6*4, *10, DRD3 rs324026 (C allele), DRD3 rs6280 (C allele), HTR2A rs6313 (TT genotype) and ANKS1B rs7968606 (T allele) will predict the high risk of intolerance to antipsychotics in adolescents with acute psychotic episodes.

**Disclosure:**

No significant relationships.

